# A Novel Ultra-High Voltage Direct Current Line Fault Diagnosis Method Based on Principal Component Analysis and Kernel Density Estimation

**DOI:** 10.3390/s25030642

**Published:** 2025-01-22

**Authors:** Haojie Zhang, Qingwu Gong

**Affiliations:** School of Electrical Engineering and Automation, Wuhan University, Wuhan 430072, China; 2020102070019@whu.edu.cn

**Keywords:** UHVDC, PCA, KDE, multiple features, line protection

## Abstract

As renewable energy resources are increasingly deployed on a large scale in remote areas, their share within the power grid continues to expand, rendering direct current (DC) transmission essential to the stability and efficiency of power systems. However, existing transmission line protection principles are constrained by limited fault feature quantities and insufficient correlation exploration among features, leading to operational refusals under remote and high-resistance fault conditions. To address these limitations in traditional protection methods, this study proposes an innovative single-ended protection principle based on Principal Component Analysis (PCA) and Kernel Density Estimation (KDE). Initially, PCA is employed for multidimensional feature extraction from fault data, followed by KDE to construct a joint probability density function of the multidimensional fault features, allowing for fault type identification based on the joint probability density values of new samples. In comparison to conventional methods, the proposed approach effectively uncovers intrinsic correlations among multidimensional features, integrating them into a comprehensive feature set for fault diagnosis. Simulation results indicate that the method exhibits robustness across various transition resistances and fault distances, demonstrates insensitivity to sampling frequency, and achieves 100% accuracy in fault identification across sampling time windows of 0.5 ms, 1 ms, and 2 ms.

## 1. Introduction

In response to the global initiative for energy conservation and emission reduction, China is advancing the construction of several large-scale wind and photovoltaic power bases, strategically situated in desert, Gobi, and arid regions [1]. These renewable energy bases, each with planned installed capacities exceeding 10 gigawatts, are positioned far from primary load centers. Ultra-high voltage direct current (UHVDC) transmission, due to its superior capacity for long-distance transmission, high throughput, and low losses, is anticipated to play a critical role in the efficient integration and delivery of renewable energy [2,3,4]. However, the complex operational environments of UHVDC lines, coupled with diverse fault types, impose significant challenges on protection systems, particularly regarding reliability, accuracy, and rapid response capabilities [5,6,7].

Traveling wave protection, as the primary protection for transmission lines, is significantly affected by transition resistance and fault distance, leading to potential failure to operate during high-resistance and remote faults [8,9]. Numerous researchers have investigated these challenges. For instance, in [10], a non-unit ultra-high-speed DC line protection method based on the first peak time was proposed, leveraging the differences in line mode fault component voltages and their first peak times at the line ends during internal and external faults in a high-voltage DC grid. In [11], an improved traveling wave protection scheme was introduced, which compensates for the fault voltage line mode components by analyzing the relationship between line mode components and fault resistance under different fault conditions. While these approaches have indeed enhanced the reliability of protection to some extent, they have not fully resolved the issue of incorrect protection operation. The propagation of fault traveling waves is influenced by various factors, including transition resistance and fault distance. Current protection principles, which typically rely on single-feature judgment, fail to comprehensively account for the impact of these complex factors.

The more comprehensive the feature information extracted from fault data, the greater the precision in fault characterization, thereby enhancing the accuracy of fault diagnosis. Principal Component Analysis (PCA), a classical dimensionality reduction technique introduced by Karl Pearson in 1901, is fundamentally designed to map high-dimensional data onto a lower-dimensional subspace while maximizing the variance captured within that subspace [12]. When applying PCA to multiple fault samples of the same type, the method effectively constructs a new coordinate system composed of principal components. These components are designed to capture the maximum variance in the data, thus providing the most informative representation of the dataset. Through this process, several key directions, known as principal components, are extracted from the high-dimensional data. These components represent the main patterns of variation within the data and serve as an alternative expression of fault characteristics [13].

Distinct fault characteristics capture fault information from multiple perspectives. The greater the number of utilized features, the more accurately the actual fault condition can be characterized, thereby enhancing protection reliability through feature complementarity. For instance, the article [14] introduces a hybrid protection scheme combining boundary protection and traveling wave protection based on stationary wavelet transform, while the article [15] proposes a pilot protection method based on transient energy ratios, leveraging the differences in transient energy within specific frequency bands at rectifier and inverter boundaries during internal and external faults. Although these methods improve protection reliability, they primarily employ a straightforward aggregation of multiple fault features, overlooking the intrinsic interrelationships among them. A simple accumulation of features lacks the capacity to capture the comprehensive nature of fault characteristics; only through an organic integration of these features into a cohesive representation can the fault’s true state be accurately conveyed.

The intricate interdependencies among fault characteristics often render traditional theoretical analysis methods inadequate for comprehensive evaluation. Kernel Density Estimation (KDE), a non-parametric statistical method, offers a solution by constructing a joint probability density distribution of multiple fault features without requiring prior assumptions about the data distribution [16]. This allows KDE to capture the intricate distribution patterns of fault characteristics. Compared to traditional probability distribution estimation methods, KDE demonstrates significant advantages in handling data with nonlinear and complex distributions [17,18].

To address these challenges, this paper introduces an advanced UHVDC line protection method combining Principal Component Analysis (PCA) and Kernel Density Estimation (KDE). Initially, an electromagnetic transient model is employed to generate a comprehensive set of fault samples. Subsequently, PCA is utilized for dimensionality reduction, extracting principal components that retain maximal information from the original data to serve as fault features. Finally, KDE estimates the joint probability density distribution function (JPDF) of these fault features, enabling fault type identification based on the probability values associated with sample positioning within this distribution. Importantly, this method relies exclusively on local measurements, thereby removing the need for communication links and markedly enhancing both the reliability and practicality of the approach. Simulation results indicate that the method is robust against variations in transition resistance and fault distance and remains insensitive to sampling frequency. This method consistently achieves 100% fault type identification accuracy across sampling time windows of 0.5 ms, 1 ms, and 2 ms, offering a novel and dependable technical solution for the secure operation of UHVDC transmission lines.

## 2. Multidimensional Fault Feature Extraction Utilizing PCA

### 2.1. Information Completeness and Fault Diagnosis

UHVDC line protection demands exceptionally rapid response times, requiring fault identification and protection actions to be executed within extremely short time intervals. Consequently, primary protection for UHVDC transmission lines predominantly employs single-ended protection schemes due to their inherent speed advantages. However, the fault diagnosis process is significantly complicated by factors such as fault resistance and fault location. Reliance solely on single-ended information within such constrained timeframes poses substantial challenges to diagnostic accuracy. The insufficient extraction and utilization of fault characteristics embedded in the acquired data may lead to diagnostic errors, thereby undermining the reliability and effectiveness of the protection system.

According to Shannon’s information theory, the accuracy of a fault diagnosis system depends on the total information content of the input signals. The mathematical expression for information content is as follows:

(1) $$I=H\left(F\right)-H\left(F\left|X\right.\right)$$

In the equation,

- *I* represents the effective information content contributed by the input signals *X* to the fault diagnosis process.
- H(F) denotes the inherent uncertainty associated with the fault state in the absence of any input information.
- H(F|X) represents the residual uncertainty of the fault state after incorporating the input information *X*.

According to Shannon’s information theory, the accuracy of fault diagnosis improves as the amount of fault characteristic information increases. In UHVDC transmission systems, fault information is conveyed to the measurement terminal through variations in the positive and negative pole voltages and currents following a fault event. However, the presence of mutual inductance coupling between the positive and negative poles introduces significant complexity to the fault analysis process. To address this, phase-mode transformation is commonly utilized to convert fault components from the phase domain to the mode domain, thereby mitigating the effects of mutual inductance. From the perspective of information completeness, the phase domain and mode domain representations are equivalent in terms of the total fault information they convey:

(2) $$\left[\begin{array}{c} U_{0}\\ U_{1} \end{array}\right]=Q\left[\begin{array}{c} U_{P}\\ U_{N} \end{array}\right]$$

(3) $$\left[\begin{array}{c} I_{0}\\ I_{1} \end{array}\right]=Q\left[\begin{array}{c} I_{P}\\ I_{N} \end{array}\right]$$

Below is the typical transformation matrix:

(4) $$Q=\frac{1}{\sqrt{2}}\left[\begin{array}{cc} 1 & 1\\ 1 & -1 \end{array}\right]$$

In the equations,

- $U_{P}$ and $U_{N}$ represent the positive pole voltage and negative pole voltage, respectively.
- $U_{0}$ and $U_{1}$ represent the zero-mode and line-mode components, respectively.

### 2.2. PCA

Excessive reliance on univariate analysis poses significant risks. In univariate analysis, the covariation with other variables is explicitly ignored, which may result in the oversight of critical features [13]. Following a fault in a UHVDC transmission line, the traveling wave stage typically persists for approximately 5 milliseconds. During this interval, the traveling wave signals predominantly comprise transient high-frequency electromagnetic waves originating from the fault point. As these signals remain largely unaffected by the control system at this stage, they preserve a greater amount of the intrinsic fault characteristic information.

When the sampling frequency is set at 20 kHz, the traveling wave phase yields between 100 and 160 sampling points. The direct application of these high-dimensional data for fault diagnosis presents several substantial challenges. Firstly, high-dimensional data are prone to the “curse of dimensionality”, where the distances between data points become nearly indistinguishable, thereby degrading the performance of classification and clustering algorithms. Furthermore, high-dimensional datasets inherently contain increased levels of noise, which complicates the training of models and heightens the risk of overfitting. Additionally, processing such a large volume of sampling points significantly increases computational complexity, potentially causing diagnostic delays, especially in real-time applications. Therefore, to optimize fault diagnosis, it is imperative to implement dimensionality reduction techniques, which can distill the most salient information from the data, thereby simplifying the analytical process and enhancing both diagnostic accuracy and efficiency.

To address the complexity associated with high-dimensional data in fault diagnosis, Principal Component Analysis (PCA) offers an effective solution as a classical dimensionality reduction technique. By applying a linear transformation, PCA maps high-dimensional data onto a lower-dimensional subspace, extracting the most significant variance information in the data. This process simplifies the analysis, reduces the influence of noise, and enhances both the accuracy and efficiency of fault diagnosis.

When PCA is applied to multiple fault samples simultaneously, it effectively constructs a new coordinate system composed of principal components. As analyzed in Section 2.1, all fault information is encapsulated within the zero-mode voltage, zero-mode current, line-mode voltage, and line-mode current. Therefore, these four physical quantities for each sample are used as the raw data for PCA processing, and the resulting principal components are utilized as the new representation of the sample. The projections of the data onto these principal components maximize the explained variance across the dataset. The principal components of the same order explain the same amount of variance in each sample, collectively capturing the primary variation characteristics of the samples. In this context, if all samples correspond to the same type of fault, each principal component can be viewed as the projection of the fault characteristics in a specific direction, with the significant principal components collectively defining the fault feature space.

Let $x=\left[x_{1},x_{2},\cdots ,x_{n}\right]$ be a vector variable with *n* components and

(5) $$X=\left[\begin{array}{cccc} X_{11} & X_{12} & \cdots & X_{1n}\\ X_{21} & X_{22} & \cdots & X_{2n}\\ \vdots & \vdots & \ddots & \vdots \\ X_{m1} & X_{m2} & \cdots & X_{mn} \end{array}\right]$$

be the sample matrix of *x*.

Where each row $X_{i}=\left[X_{i1},X_{i2},\cdots ,X_{in}\right]$ represents the data for the *i*th sample, i=1,2,⋯,m, and *m* is the total number of samples; each column $X_{j}=\left[X_{1j},X_{2j},\cdots ,X_{mj}\right]$ represents the data for the *j*th component, j=1,2,⋯,n, and *n* is the total number of components.

For each component $X_{j}$, calculate its mean $\mu_{j}$:

(6) $$\mu_{j}=\frac{1}{m}\sum_{i=1}^{m}X_{ij}$$

Next, calculate the standard deviation $\sigma_{j}$ for each component $X_{j}$:

(7) $$\sigma_{j}=\sqrt{\frac{1}{n}\sum_{i=1}^{m}{\left(X_{ij}-\mu_{j}\right)}^{2}}$$

Then, standardize each component $X_{j}$ in the original matrix to obtain the standardized matrix *Z*:

(8) $$Z_{ij}=\frac{X_{ij}-\mu_{j}}{\sigma_{j}}$$

Afterward, calculate the covariance matrix of the standardized data:

(9) $$Cov\left(Z\right)=\frac{1}{m-1}Z^{T}Z$$

where Cov(Z) is the covariance matrix of *Z*.

After that, perform eigenvalue decomposition on the covariance matrix to obtain the eigenvalues and eigenvectors:

(10) Cov(z)·v=λ·v

In which *v* is an eigenvector and λ is its corresponding eigenvalue.

Then, rank the eigenvalues in descending order and select the top *k* eigenvectors corresponding to the largest eigenvalues. These top *k* principal components capture the most significant variance in the data:

(11) $$V_{k}=\left[v_{1},v_{2},\ldots ,v_{k}\right]$$

Finally, transform the original data into the new subspace defined by the selected principal components:

(12) $$Z_{PCA}=Z\cdot V_{k}$$

where $Z_{PCA}$ is the transformed data matrix.

### 2.3. PCA-Based Fault Characteristics

Current protection algorithms generally limit fault feature selection to one or two indicators, such as traveling wave and voltage surge protection. However, due to the incomplete extraction of fault waveform characteristics, these protection methods may struggle to accurately discriminate between internal and external faults under extreme conditions, including remote or high-resistance faults.

Principal Component Analysis (PCA) facilitates dimensionality reduction by projecting data along directions of maximal variance, thus preserving the essential structure of the dataset. In high-dimensional spaces, many features may exhibit substantial linear correlations, leading to redundancy. PCA addresses this by identifying a set of orthogonal principal components, projecting the original data onto these uncorrelated directions to retain the fundamental fault characteristics. By selecting only the most significant principal components, PCA effectively mitigates noise and concentrates the data along the most representative feature axes, thereby enhancing the robustness of fault detection.

**Figure 1:**
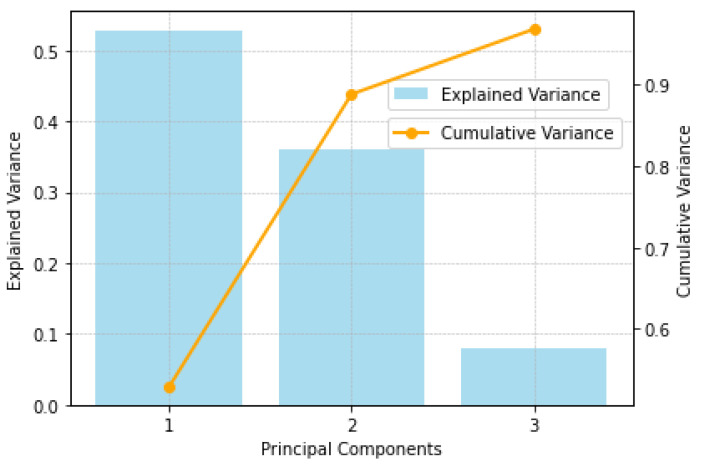
Explained and cumulative variance of principal components.

**Figure 2:**
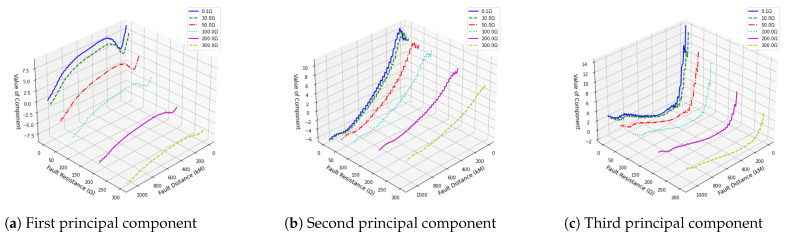
Principal components comparison across different fault resistances and distances.

Applying PCA to feature extraction for positive pole grounding fault samples under varying transition resistances and fault distances, as illustrated in Figure 1, shows that the first three principal components capture over 97% of the variance. This finding indicates that these three components alone retain more than 97% of the original data information, allowing dimensionality reduction with minimal information loss. Additionally, the variation trends of these three principal components in relation to transition resistance and fault distance, as depicted in Figure 2, reveal consistent patterns across different transition resistances. This analysis confirms that the principal components derived from PCA effectively encapsulate the multidimensional nature of fault characteristics.

## 3. Protection Scheme Based on KDE Joint Probability Density Distribution

### 3.1. KDE

The relationships among fault features are often inherently complex, and without thorough exploration and utilization of their intrinsic correlations, a simple aggregation of these features may markedly compromise the accuracy of fault diagnosis. Integrating multidimensional features into a cohesive analytical framework allows for a more precise representation of the fault’s true state, thereby enhancing diagnostic accuracy. Although Principal Component Analysis (PCA) is effective for dimensionality reduction and preserves essential data information, the resulting principal components lack explicit physical interpretation, and their intrinsic interrelations remain challenging to elucidate through conventional theoretical methods.

To address the complexity of inter-feature relationships, modeling and analyzing the joint distribution of multiple fault features via multivariate statistical methods has emerged as a potent solution. Kernel Density Estimation (KDE), a non-parametric statistical approach, is introduced to achieve a deeper understanding and analysis of these complex feature interactions. KDE estimates the probability density function of data, capturing intricate inter-feature dependencies without requiring an a priori assumption about the distribution form. Specifically, KDE treats each data point as the center of a kernel function, estimating the overall probability density through weighted summation across all kernels. Common kernels include Gaussian, uniform, and triangular functions. This method effectively uncovers the joint distribution of multiple fault features, providing a robust foundation for achieving high-precision fault diagnosis.

For a dataset comprising *n* samples $\left\{y_{1},y_{2},\cdots ,y_{n}\right\}$, each characterized by *d* variables, the mathematical formulation of KDE is expressed as follows:

(13) $$\hat{f}\left(y\right)=\frac{1}{nh^{d}}\sum_{i=1}^{n}K(\frac{y-y_{i}}{h})$$

where $\hat{f}\left(y\right)$ is the estimated probability density function at the point *y*. $y=(y_{1},y_{2},\cdots ,y_{d})$ represents the coordinates of the point where the density is being estimated, with *d* dimensions. *n* is the number of samples. *h* is the bandwidth parameter, which controls the smoothness of the kernel function. K(·) is the kernel function, with common choices including the Gaussian kernel, uniform kernel, and triangular kernel.

The bandwidth *h* in KDE is a pivotal parameter that directly influences the quality of the density estimate. An undersized bandwidth may lead to an excessively variable estimate, rendering it highly sensitive to data fluctuations, whereas an oversized bandwidth may result in an overly smoothed estimate that obscures critical features within the data. Common approaches for optimal bandwidth selection include the Rule of Thumb, which provides an empirical calculation based on sample standard deviation and sample size, and Cross-Validation, an optimization method that determines the bandwidth by balancing the bias–variance trade-off, thereby achieving a more reliable density estimation.

### 3.2. Joint Probability Density Distribution of Fault Features Based on KDE

Given that the probability density distribution of faults is inherently multivariate, a single feature is insufficient to accurately capture the true fault distribution. However, applying Kernel Density Estimation (KDE) to high-dimensional data entails significant challenges, such as high computational complexity, data sparsity, difficulties in bandwidth selection, the curse of dimensionality, and visualization constraints. As previously analyzed, the first three principal components retain over 97% of the original data information, making them suitable for KDE in this study.

Prior to implementing KDE, an appropriate kernel function and bandwidth parameter must be selected. The choice of kernel function depends on the characteristics of the data while the bandwidth parameter determines the kernel’s width, thereby directly affecting the smoothness of the density estimate. This study adopts a Gaussian kernel function, though there is no universally optimal method for selecting bandwidth. After extensive trials, a bandwidth of 0.085, which demonstrated the best performance, was chosen.

### 3.3. Fault Diagnosis Based on Joint Probability Density Distribution

#### 3.3.1. Start-Up Module

The protection start-up module is designed to detect fault signals on transmission lines and activate subsequent protection mechanisms. To mitigate the risk of false triggering due to noise interference and outliers, this study utilizes the mean absolute value of the line voltage difference as the criterion for protection initiation. The specific formulation is presented as follows:

(14) $$\left|\sum_{i=0}^{n}U_{i}-\sum_{j=1}^{n+1}U_{j}\right|>\Delta U$$

where $U_{p,t}$ and $U_{p,t+1}$ represent the *t*th and t+1th points in the fault recording, respectively. t=1,2,⋯k−1, let *k* denote the total length of the fault recording. Let ΔU represent the threshold value, which in this study is set to $0.01U_{f}$.

#### 3.3.2. Fault Diagnosis Unit

**Figure 3:**
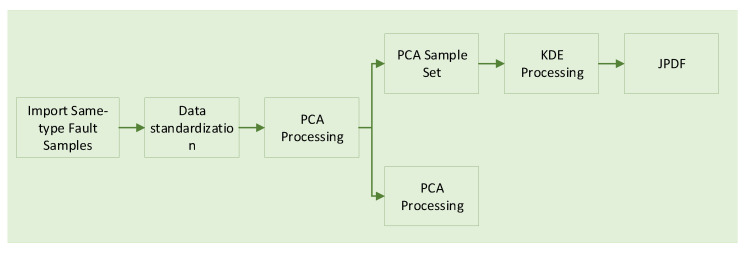
Flowchat for joint probability density function estimation based on PCA and KDE.

According to the flowchart in Figure 3, the joint probability density function for an individual fault type can be derived. This distribution represents the probability of normal data occurrence across different regions. In high-density regions, the probability of observing normal data points is elevated, whereas in low-density regions, this probability is significantly diminished. To assess the anomaly of new data, a density threshold can be established, typically selected as a lower percentile of the normal data density distribution. Regions with densities below this threshold are thereby classified as anomalous, reflecting an extremely low probability of normal data appearing in these areas. For each new data point, its probability density within the established normal data distribution is computed; if this density value falls below the defined threshold, the data point is classified as anomalous; otherwise, it is considered to be within the normal range.

(15) $$Output_{i}\left(x\right)=\left\{\begin{array}{c} 1,\;if\;JPDF_{i}\left(x\right)\geq \delta_{i}\\ 0,\;if\;JPDF_{i}\left(x\right)<\delta_{i} \end{array}\right.$$

where $Output_{i}$ denotes the output of the fault diagnostic unit, where values of 0 and 1 represent normal and fault conditions, respectively. The index *i* assumes values of one, two, and three, corresponding to positive pole grounding fault, negative pole grounding fault, and bipolar grounding fault, respectively. JDPF represents the Joint Density Probability Function and δ represents the 1% density threshold.

#### 3.3.3. Multi-Type Fault Diagnosis

After deriving the JPDF for positive pole grounding faults, negative pole grounding faults, and bipolar grounding faults, multi-type fault diagnosis within the line region can be effectively conducted.

**Figure 4:**
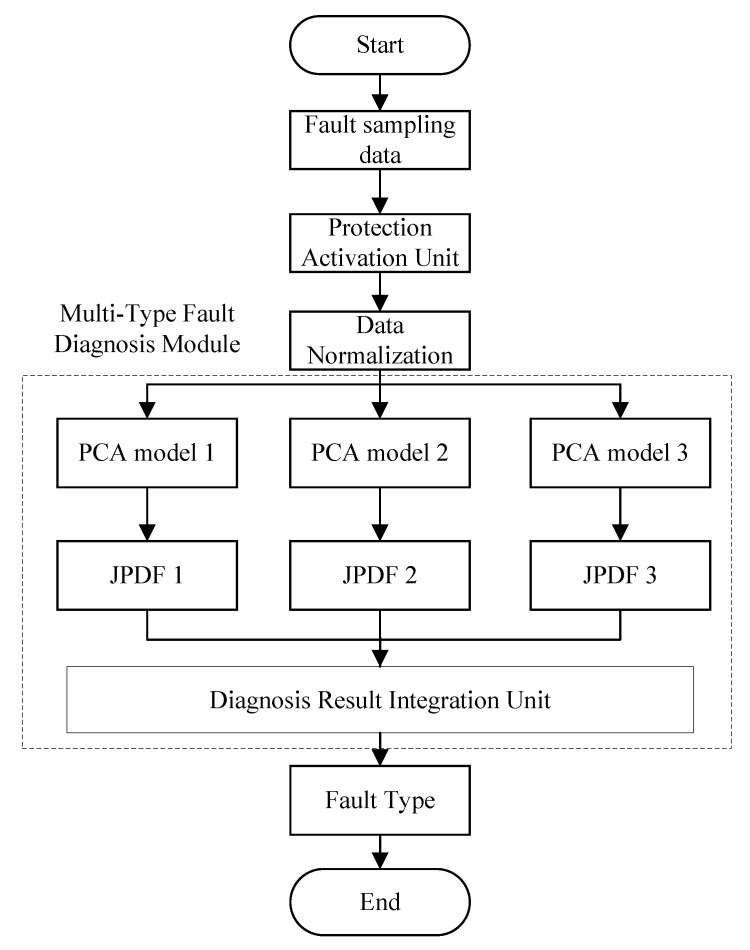
Flowchart for multi-fault type diagnosis of UHVDC transmission lines based on PCA and KDE.

As illustrated in Figure 4, the flowchart for multi-type fault diagnosis in UHVDC transmission lines is as follows: once newly acquired data meet the threshold set by the protection activation unit, they are standardized and fed into the multi-type fault diagnosis module (MTFDM), which subsequently outputs the identified fault type. This module comprises three PCA models and three joint probability density functions (JPDFs), corresponding to positive pole grounding fault, negative pole grounding fault, and bipolar grounding fault. The output values of these JPDFs are then passed to the diagnostic result integration unit to derive the final fault diagnosis.

**Figure 5:**
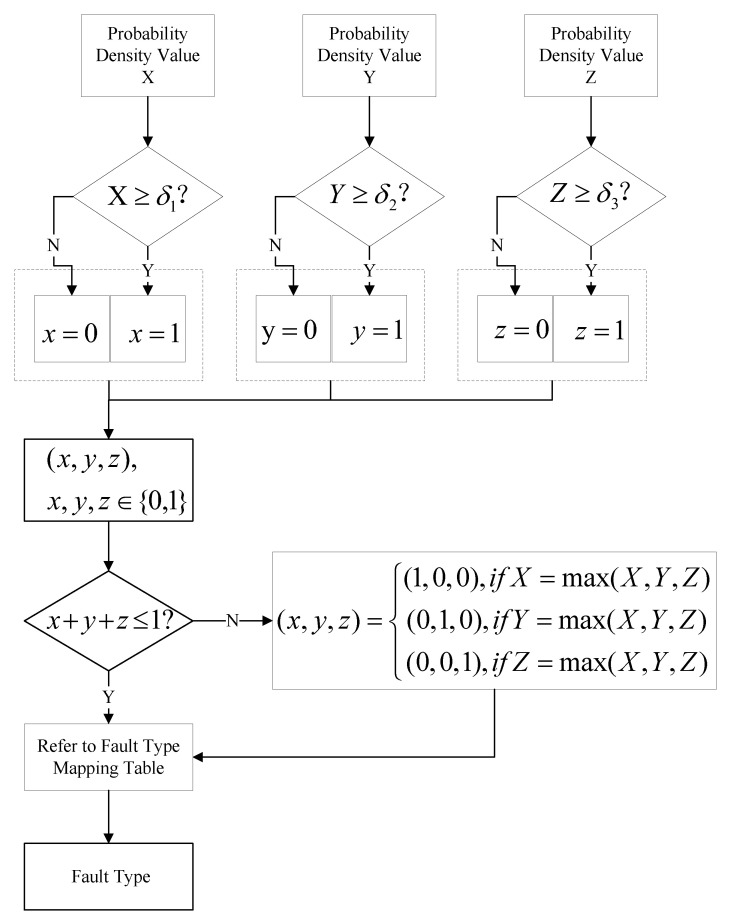
Fault diagnosis integration unit.

The diagnostic result integration process is detailed in Figure 5. Specifically, the three JPDF values are individually compared against their respective thresholds. If a value exceeds its threshold, the output is set to 1; otherwise, it is set to 0. These outputs form a three-dimensional vector containing only 0 s and 1 s. The fault type is then determined by referencing the fault type mapping table (Table 1). Under normal conditions, this vector contains at most one value of 1, resulting in the following interpretations: (1, 0, 0) for positive pole grounding fault, (0, 1, 0) for negative pole grounding fault, (0, 0, 1) for bipolar grounding fault, and (0, 0, 0) for non-line internal fault.

In cases where the vector contains two or more values of 1, the initial JPDF values are compared, and the maximum value is set to 1, with the remaining values set to 0. The fault type is then determined by referencing the fault type mapping table.

## 4. Simulation Validation

### 4.1. UHVDC Simulation Model and Fault Sample Generation

**Figure 6:**
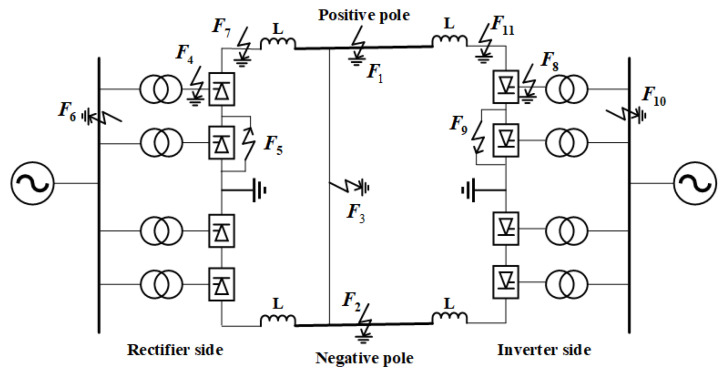
Topology of UHVDC transmission system.

The ±800 kV UHVDC transmission system, as depicted in Figure 6, has been rigorously modeled in PSCAD. This model integrates essential components, including a rectifier station, an inverter station, and a 1085 km long DC transmission line. The core functionalities of the modeled system closely replicate those of the actual engineering infrastructure. Moreover, the dynamic performance simulations conducted using this model are in strong agreement with empirical results obtained from real-world engineering trials, thereby validating the model’s accuracy and reliability in representing the operational characteristics of the UHVDC transmission system.

In Table 2, the fault types within the line area are designated as F1, F2, and F3. Transition resistances are categorized into six levels: 0.1 Ω, 5 Ω, 50 Ω, 100 Ω, 200 Ω, and 300 Ω. Fault locations are systematically chosen at intervals of 10 km, extending from the rectifier side to the inverter side. Table 3 outlines the external fault types (EF), labeled F4 through F11, which are strategically positioned in various regions on both the rectifier and inverter sides. These external faults are also evaluated across the same six levels of transition resistance: 0.1 Ω, 5 Ω, 50 Ω, 100 Ω, 200 Ω, and 300 Ω.

Simulations were conducted using the parameters specified in Table 2 and Table 3, resulting in the generation of 648 samples each for positive pole ground faults, negative pole ground faults, and bipolar ground faults, along with 48 samples for external line faults. The analysis will proceed with a focus on positive pole ground faults as a representative case. The 648 fault samples were first subjected to preprocessing, followed by a PCA to explore the underlying data structure.

### 4.2. Internal and External Fault Diagnosis

To evaluate the efficacy of the proposed method, data within a 1ms time window following the activation of the fault initiation module were selected. The fault samples from Section 4.1 were preprocessed in accordance with the procedure outlined in Figure 3, and joint probability density functions (JPDFs) for positive pole grounding faults (JPDF1), negative pole grounding faults (JPDF2), and bipolar grounding faults (JPDF3) were subsequently fitted. Thereafter, all samples were processed through the diagnostic framework depicted in Figure 4, with their probability density values under various JPDFs summarized in Table 4. As presented in Table 4, the probability density values for non-corresponding fault types are significantly lower than the established thresholds in most scenarios, whereas the probability density values for corresponding fault types consistently exceed these thresholds. In rare instances where the probability density values of non-corresponding fault types surpass the thresholds, accurate identification is still achieved using the MTFDM method. Furthermore, external faults consistently yield probability density values significantly below the thresholds across all three joint probability density functions (JPDFs). As demonstrated in Table 5, the proposed method achieves 100% accuracy in fault type identification, highlighting its robustness and reliability.

### 4.3. Impact of Fault Distance and Transition Resistance

The fault identification results presented in Section 4.2 demonstrate that the proposed method achieves 100% accuracy in identifying faults across the entire line length under a 300 Ω transition resistance. To further assess the robustness of this method, additional identification tests were conducted for faults at various line locations under transition resistances of 400 Ω and 500 Ω. The results are shown in Table 6.

### 4.4. Impact of Time Window

To assess the impact of data time window length on the performance of the proposed method, tests were conducted using time windows of 0.5 ms (10 sampling points) and 2 ms (40 sampling points). The results, presented in Table 7 and Table 8, demonstrate that the fault diagnosis accuracy reached 100% with time windows of both 0.5 ms and 1 ms, highlighting the method’s ability to maintain high diagnostic precision even under shorter time windows.

### 4.5. Impact of Sampling Frequency

To assess the performance of the proposed method across different sampling frequencies, the original fault data (20 kHz) was downsampled to generate fault samples at 10 kHz and 5 kHz. Tests were then conducted using the proposed method on these samples, with the results presented in Table 9 and Table 10. The findings indicate that even at a reduced sampling frequency of 10 kHz, the method maintains a fault identification accuracy of 100%. Furthermore, at a sampling frequency of 5kHz, the average fault identification accuracy remains above 99%, underscoring the method’s robustness for sampling frequency variations.

## 5. Conclusions

This paper introduces an advanced protection principle based on Principal Component Analysis (PCA) and Kernel Density Estimation (KDE). PCA is utilized to extract multidimensional features from fault signals, capturing the primary variance structure within the data. Subsequently, KDE models the joint probability density distribution of these features, constructing a comprehensive joint probability density function that encompasses positive pole grounding, negative pole grounding, and bipolar grounding faults. This method efficiently integrates and leverages multiple fault characteristics, significantly improving the comprehensiveness and precision of fault diagnosis. Notably, the approach relies exclusively on single-end measurements and consistently achieves 100% fault diagnosis accuracy across the entire line, even under high fault resistance conditions of up to 300 Ω. Experimental evaluations reveal that the proposed method exhibits robustness to sampling frequency, maintaining a superior diagnostic accuracy even at a sampling rate of 10 kHz. Moreover, the method’s robustness is further substantiated through experiments conducted under varying computational time windows, consistently achieving 100% accurate fault diagnosis within 0.5 ms across different window conditions.

## Figures and Tables

**Table 1:** Fault type mapping table.

Fault Sequence Number	Fault Type
(1, 0, 0)	Positive pole ground fault
(0, 1, 0)	Negative pole ground fault
(0, 0, 1)	Bipole ground fault
(0, 0, 0)	Non-line internal fault

**Table 2:** Simulation traversal parameter table for UHVDC line faults.

Parameters	Value
Fault type	F1, F2, F3
Fault resistance (Ω)	0.1, 10, 50, 100, 200, 300
Fault location (kM)	10, 20, 30, ..., 1080

**Table 3:** Simulation traversal parameter table for external faults in UHVDC transmission line.

Parameters	Value
Fault type	F4, F5, F6, F7, F8, F9, F10, F11
Fault resistance (Ω)	0.1, 10, 50, 100, 200, 300

**Table 4:** Statistical table of fault sample probability density values across different joint probability density functions.

Fault Type	JPDF1			JPDF2			JPDF3		
	**Min**	**Max**	δ **1**	**Min**	**Max**	δ **2**	**Min**	**Max**	δ **3**
PG	$7.4884\times 10^{-1}$	$1.5354\times 10^{1}$	$6.0000\times 10^{-1}$	0.0000	$2.4595\times 10^{-5}$	N/A	0.0000	$1.9564\times 10^{-1}$	N/A
NG	0.0000	$6.2148\times 10^{-6}$	N/A	$7.4884\times 10^{-1}$	$1.4934\times 10^{1}$	$6.0000\times 10^{-1}$	0.0000	$6.3470\times 10^{-1}$	N/A
BG	0.0000	$3.3814\times 10^{-1}$	N/A	0.0000	$1.7808\times 10^{-1}$	N/A	$7.4884\times 10^{-1}$	9.5603	$6.0000\times 10^{-1}$
EF	0.0000	$5.1508\times 10^{-4}$	N/A	0.0000	$1.3137\times 10^{-51}$	N/A	0.0000	$2.6370\times 10^{-6}$	N/A

**Table 5:** Fault identification accuracy for joint probability density functions and the proposed fault diagnosis method.

Fault Type	Acc of JPDF1 (%)	Acc of JPDF2 (%)	Acc of JPDF3 (%)	Acc of MTFDM (%)
PG	100%	100%	100%	100%
NG	100%	100%	99.85%	100%
BG	100%	100%	100%	100%
EF	100%	100%	100%	100%

**Table 6:** Statistical analysis of fault sample probability density values across various joint probability density functions within a 0.5 ms time window.

No.	Actual Fault Type	Fault Resistance (Ω)	Fault Distance	JPDF1	JPDF2	JPDF3	Threshold	Diagnosed Fault Type
1	F1	400	10	3.4824	0.0000	$8.4962\times 10^{-13}$	$5.9358\times 10^{-1}$	F1
2	F1	400	500	3.5787	$6.9955\times 10^{-14}$	$2.2144\times 10^{-6}$	$5.9358\times 10^{-1}$	F1
3	F1	400	1000	$4.8691\times 10^{1}$	$6.8574\times 10^{-63}$	$1.0902\times 10^{-50}$	$5.9358\times 10^{-1}$	F1
4	F1	500	10	3.4824	0.0000	$2.6440\times 10^{-13}$	$5.9358\times 10^{-1}$	F1
5	F1	500	500	3.4839	$4.8921\times 10^{-53}$	$1.0143\times 10^{-7}$	$5.9358\times 10^{-1}$	F1
6	F1	500	1000	$3.9171\times 10^{1}$	$1.07438\times 10^{-15}$	$4.9540\times 10^{-53}$	$5.9358\times 10^{-1}$	F1
7	F2	400	10	0.0000	3.4824	$3.5457\times 10^{-13}$	$5.9358\times 10^{-1}$	F2
8	F2	400	500	$2.1279\times 10^{-25}$	3.6080	$1.3813\times 10^{-14}$	$5.9358\times 10^{-1}$	F2
9	F2	400	1000	$7.5518\times 10^{-72}$	$4.9586\times 10^{1}$	$2.2550\times 10^{-1}$	$5.9358\times 10^{-1}$	F2
10	F2	500	10	0.0000	3.4824	$4.6923\times 10^{-13}$	$5.9358\times 10^{-1}$	F2
11	F2	500	500	$1.2097\times 10^{-53}$	3.4841	$3.1743\times 10^{-83}$	$5.9358\times 10^{-1}$	F2
12	F2	500	1000	$1.4720\times 10^{-72}$	$3.8881\times 10^{1}$	$5.7233\times 10^{-226}$	$5.9358\times 10^{-1}$	F2
13	F3	400	10	$5.6972\times 10^{-60}$	$2.1766\times 10^{-23}$	$2.1867\times 10^{1}$	$5.9358\times 10^{-1}$	F3
14	F3	400	500	$8.9410\times 10^{-76}$	$5.7646\times 10^{-15}$	$1.4685\times 10^{1}$	$5.9358\times 10^{-1}$	F3
15	F3	400	1000	$8.5810\times 10^{-86}$	$3.7687\times 10^{-39}$	$1.5757\times 10^{1}$	$5.9358\times 10^{-1}$	F3
16	F3	500	10	$1.2439\times 10^{-46}$	$8.2321\times 10^{-17}$	$1.5794\times 10^{1}$	$5.9358\times 10^{-1}$	F3
17	F3	500	500	$4.9941\times 10^{-76}$	$3.6909\times 10^{-14}$	8.5017	$5.9358\times 10^{-1}$	F3
18	F3	500	1000	$2.7900\times 10^{-89}$	$3.0327\times 10^{-43}$	$1.670\times 10^{1}$	$5.9358\times 10^{-1}$	F3

**Table 7:** Fault identification accuracy table for joint probability density functions and the proposed fault diagnosis method within a 2 ms time window.

Fault Type	Acc of JPDF1 (%)	Acc of JPDF2 (%)	Acc of JPDF3 (%)	Acc of MTFDM (%)
PG	100%	100%	100%	100%
NG	100%	100%	100%	100%
BG	100%	100%	100%	100%
EF	100%	100%	100%	100%

**Table 8:** Fault identification accuracy table for joint probability density functions and the proposed fault diagnosis method within a 0.5 ms time window.

Fault Type	Acc of JPDF1 (%)	Acc of JPDF2 (%)	Acc of JPDF3 (%)	Acc of MTFDM (%)
PG	100%	100%	98.61%	100%
NG	100%	100%	100%	100%
BG	99.85%	100%	100%	100%
EF	100%	100%	100%	100%

**Table 9:** Fault identification accuracy table for joint probability density functions and the proposed fault diagnosis method at a sampling frequency of 5 kHz.

Fault Type	Acc of JPDF1 (%)	Acc of JPDF2 (%)	Acc of JPDF3 (%)	Acc of MTFDM (%)
PG	100%	100%	100%	100%
NG	100%	100%	99.69%	99.85%
BG	100%	100%	100%	100%
EF	100%	100%	100%	100%

**Table 10:** Fault identification accuracy table for joint probability density functions and the proposed fault diagnosis method at a sampling frequency of 10 kHz.

Fault Type	Acc of JPDF1 (%)	Acc of JPDF2 (%)	Acc of JPDF3 (%)	Acc of MTFDM (%)
PG	100%	100%	100%	100%
NG	100%	100%	100%	100%
BG	100%	100%	100%	100%
EF	100%	100%	100%	100%

## Data Availability

The data presented in this study are available upon request from the corresponding author. The data are not publicly available due to confidentiality.

## Abbreviations

The following abbreviations are used in this manuscript:

DC	Direct current
PCA	Principal Component Analysis
KDE	Kernel Density Estimation
UHVDC	Directory of open-access journals
